# Association of Diet With Erectile Dysfunction Among Men in the Health Professionals Follow-up Study

**DOI:** 10.1001/jamanetworkopen.2020.21701

**Published:** 2020-11-13

**Authors:** Scott R. Bauer, Benjamin N. Breyer, Meir J. Stampfer, Eric B. Rimm, Edward L. Giovannucci, Stacey A. Kenfield

**Affiliations:** 1 Department of Medicine, University of California, San Francisco; 2 Department of Urology, University of California, San Francisco; 3 Division of General Internal Medicine, San Francisco VA Medical Center, San Francisco, California; 4 Department of Epidemiology and Biostatistics, University of California, San Francisco; 5 Departments of Nutrition & Epidemiology, Harvard T.H. Chan School of Public Health, Boston, Massachusetts; 6 Channing Division of Network Medicine, Department of Medicine, Brigham and Women’s Hospital, Boston, Massachusetts

## Abstract

**Importance:**

Erectile dysfunction, especially in younger men, is an early sign of cardiovascular disease and may decrease quality of life. Men may be motivated to adopt a healthy dietary pattern if it lowers their risk of erectile dysfunction.

**Objective:**

To assess the association between adherence to a diet quality index based on healthy dietary patterns and erectile dysfunction in men.

**Design, Setting, and Participants:**

This population-based prospective cohort study included men from the Health Professionals Follow-up Study with follow-up from January 1, 1998, through January 1, 2014. Participants included US male health professionals aged 40 to 75 years at enrollment. Men with erectile dysfunction or a diagnosis of myocardial infarction, diabetes, stroke, or genitourinary cancer at baseline were excluded. Analyses were completed in February 2020.

**Exposures:**

A food frequency questionnaire was used to determine nutrient and food intake every 4 years.

**Main Outcomes and Measures:**

Diet quality was assessed by Mediterranean Diet score and the Alternative Healthy Eating Index 2010 score, with higher scores indicating healthier diet. Dietary index scores were cumulatively updated from 1986 until men developed erectile dysfunction, cardiovascular disease, died, or were lost to follow-up. Incident erectile dysfunction was assessed with questionnaires in 2000, 2004, 2008, and 2012. Hazard ratios (HRs) by prespecified categories or quintiles of dietary index scores were estimated using Cox proportional hazards regression analyses stratified by age.

**Results:**

Among 21 469 men included in analysis, mean (SD) age at baseline was 62 (8.4) years. During a mean (SD) follow-up of 10.8 (5.4) years and 232 522 person-years, there were 968 incident erectile dysfunction cases among men younger than 60 years, 3703 cases among men aged 60 to less than 70 years, and 4793 cases among men aged 70 years or older. Men younger than 60 years and in the highest category of the Mediterranean Diet score had the lowest relative risk of incident erectile dysfunction compared with men in the lowest category (HR, 0.78; 95% CI, 0.66-0.92). Higher Mediterranean diet scores were also inversely associated with incident erectile dysfunction among older men (age 60 to <70 years: HR, 0.82; 95% CI, 0.76-0.89; age ≥70 years: HR, 0.93; 95% CI, 0.86-1.00). Men scoring in the highest quintile of the Alternative Healthy Eating Index 2010 also had a lower risk of incident erectile dysfunction, particularly among men age younger than 60 years (quintile 5 vs quintile 1: HR, 0.78; 95% CI, 0.63-0.97).

**Conclusions and Relevance:**

This cohort study found that adherence to healthy dietary patterns was associated with a lower risk for erectile dysfunction, suggesting that a healthy dietary pattern may play a role in maintaining erectile health.

## Key Points

**Question:**

Is diet quality associated with risk of erectile dysfunction?

**Findings:**

In this cohort study among 21 469 men in the Health Professionals Follow-up Study, higher diet quality based on adherence to either a Mediterranean or Alternative Healthy Eating Index 2010 diet, which emphasize the consumption of vegetables, fruits, nuts, legumes, and fish or other sources of long-chain (n-3) fats, as well as avoidance of red and processed meats, was found to be associated with a lower risk of developing erectile dysfunction.

**Meaning:**

These findings suggest that a healthy dietary pattern may play a role in maintaining erectile function in men.

## Teaser

This cohort study examines the association of adherence to Mediterranean or Alternative Healthy Eating Index 2010 diets with incidence of erectile dysfunction in men.

## Introduction

Erectile dysfunction affects an estimated 18 million men in the US,^1^ with the disease burden expected to grow as the population ages. Erectile dysfunction is associated with reduced sexual intimacy and health-related quality of life as well as psychological distress for both the affected men and their sexual partners.^2,3^ If all affected men sought treatment, treatment costs in the US could reach $15 billion.^4^ Modifiable risk factors for erectile dysfunction, particularly among younger men (ie, age <60 years),^5^ are largely shared with cardiovascular disease (CVD) and include smoking, obesity, sedentary behavior, diabetes, hypertension, hyperlipidemia, and metabolic syndrome.^6,7^ In fact, erectile dysfunction is associated with future CVD and may represent an opportunity to identify and modify shared risk factors.^8^ Although evidence-based lifestyle interventions,^9^ including healthy dietary patterns,^10,11^ are offered to men interested in lowering their CVD risk, it is unknown whether healthy dietary patterns are associated with lower risk of erectile dysfunction.

Studies evaluating the association between diet and erectile dysfunction are limited, and have focused on men with diabetes or prevalent erectile dysfunction. Several small- to moderate-sized randomized clinical trials report that multimodal lifestyle and weight loss interventions improve erectile dysfunction among men with significant cardiovascular risk factors.^12^ However, fewer studies have examined the association between a healthy dietary pattern and erectile dysfunction risk,^13,14^ and to our knowledge, no prior studies have evaluated the association between adherence to healthy dietary patterns and incident erectile dysfunction in men without diabetes.

We analyzed the association of 2 dietary index scores representative of healthy dietary patterns, the Mediterranean Diet score (MDS) and the Alternative Healthy Eating Index 2010 (AHEI-2010) score, with incident erectile dysfunction in the Health Professionals Follow-up Study, a large prospective cohort study of adult men. We hypothesized that greater adherence to healthy dietary patterns would be associated with lower incident erectile dysfunction, particularly among younger men.

## Methods

This study was approved by the Human Subjects Committee at the Harvard T.H. Chan School of Public Health. As approved by the Human Subjects Committee, the return of a questionnaire was considered to imply consent. This study is reported following the Strengthening the Reporting of Observational Studies in Epidemiology (STROBE) reporting guideline.

### Participants

The Health Professionals Follow-up Study is a prospective study of US male health professionals who enrolled in 1986 by completing a mailed questionnaire. Detailed methods have been published elsewhere.^15^ Briefly, enrolled participants complete a food frequency questionnaire (FFQ) every 4 years and questionnaires that included information regarding lifestyle factors, health outcomes, and medications every 2 years (response rate, 96%).

Of 51 529 men enrolled in 1986, 5510 died prior to 1998; we excluded 8505 men who did not complete the FFQ in 1998, 2551 men who reported a diagnosis of prostate, bladder, or testicular cancer prior to 1998, 445 men who reported implausible energy intake (ie, <800 or >4200 kcal/d), 429 men who did not complete the erectile function assessment questionnaire in 2000, and 35 men who were missing age information. We further excluded 5458 men (20.3% of eligible participants) who reported erectile dysfunction prior to the first erectile function assessment in 2000. To avoid unmeasured confounding due to an unhealthy dietary pattern prior to their diagnosis, we also excluded 3796 men with a history of myocardial infarction, 2499 men with diabetes, and 832 men with a history of stroke prior to 1998 (eFigure in the Supplement).

### Assessment of Dietary Patterns

Usual dietary intake of approximately 130 food items was estimated over the previous year using a FFQ completed every 4 years starting in 1986. Participants indicated portion size and frequency of consumption, from never or less than 1 serving per month to 6 or more servings per day. This FFQ has been validated against the standard criterion of repeated 1-week diet records. The mean Pearson correlation coefficient for all foods was 0.63, and 73% of food items had correlation coefficients of 0.50 or greater.^16^ Calculation of dietary index scores using self-reported intake of specific food items with a FFQ have been previously published.^17,18^

To calculate the MDS index score, participants received 1 point each for consuming above the median intake of vegetables, legumes, fruits and nuts, grains, fish, and the ratio of polyunsaturated to saturated lipids, calculated separately for each dietary questionnaire cycle; 1 point each for consuming less than the median dairy and red or processed meat intake; and 1 point for alcohol intake between 10 and 50 g per day (total score range, 0-9). Monounsaturated fat, used in the traditional MDS,^17^ was not used for the lipid ratio because the main dietary contributor of monounsaturated fat in our cohort was beef.^19,20^ To harmonize with prior publications, the MDS was reported categorically as low (0-3), moderate (4-5), and high (6-9) adherence to a Mediterranean dietary pattern.^20^

To calculate the AHEI-2010 score, participants were scored on 11 items with predefined criteria for complete adherence vs nonadherence based on the Healthy Eating Pyramid (2010 version).^18,21^ Higher intake of fruits, vegetables, whole grains, nuts and legumes, polyunsaturated fats, and ω-3 fatty acids, and lower intake of red and processed meats, sugar-sweetened beverages, trans fatty acids, and sodium contribute to a higher (healthier) dietary index score. Moderate alcohol intake (0.5-2 drinks/d) contributes to a higher index score. Each item is scored from 0 (complete nonadherence) to 10 (complete adherence), with partial scores awarded for proportional intake (total score range: 0-110). The AHEI-2010 score was evaluated by quintile.

To examine associations with individual nutrient and food components, we analyzed each of the dietary index score components categorically using intake servings per week of MDS components and quintiles of AHEI-2010 components.

### Outcome Assessment

Starting in 2000, participants were asked to rate their current ability to maintain an erection sufficient for intercourse without treatment as very poor, poor, fair, good, or very good. Each question included a time grid with year and month increments (before 1986, 1986-1989, 1990-1994, 1995 or later, and in the past 3 months) to allow participants to report historically if and when erectile function changed. Participants were asked to report their current function (without treatment) again in 2004, 2008, and 2012. Consistent with prior studies, we defined incident erectile dysfunction as a response of poor or very poor in any periods from 2000 to 2012 among men who reported good or very good erectile function prior to 2000.^22,23^ Date of diagnosis was defined as the date of return of the questionnaire, and we censored at first report of erectile dysfunction.

### Data Analysis

Each participant contributed person-time from date of return of the 1998 questionnaire until date of first report of erectile dysfunction, genitourinary cancer (ie, prostate, bladder, or testicular), death, loss to follow-up, or end of follow-up (ie, January 1, 2014). To estimate long-term intake and reduce random within-person variation,^24^ we calculated the cumulative mean of dietary index scores from all FFQs completed prior to first report of erectile dysfunction, CVD, death, lost to follow-up, or the last FFQ in 2010. We stopped updating dietary index scores after a CVD diagnosis because CVD is a strong potential confounder of the association between MDS or AHEI-2010 and incident erectile dysfunction and dietary changes are more common after a CVD diagnosis.^25^

Multivariable adjusted Cox proportional hazards models were used to calculate hazard ratios (HRs) and 95% CIs for associations between categories of dietary index score and risk of incident erectile dysfunction. We used age in months as the time scale and stratified baseline hazard by calendar year. Final multivariable models were adjusted for biennially updated covariates that included smoking (never, past, or current 1-14, 15-24, or ≥25 cigarettes/d), body mass index (BMI, calculated as weight in kilograms divided by height in meters squared; <25, 25-29.9, or ≥30), physical activity (total metabolic equivalent task [MET]–hours/week, categorized in quintiles), incident CVD during follow-up (yes or no), incident diabetes during follow-up (yes or no), hyperlipidemia (yes or no), hypertension (yes or no), depression (yes or no), antidepressant or antipsychotic medication use (yes or no), benzodiazepine use (yes or no), α-blocker or 5α-reductase inhibitor use (yes or no), marital status (married, divorced, separated, widowed, or never married), self-reported race (White, Black, Asian, or other), and total caloric intake (kilocalories/d). If exposure or covariate data were missing for a questionnaire cycle, we carried forward nonmissing exposure and covariate data from the previous cycle. To test for a linear trend across categories of dietary index score, we modeled scores as continuous variables using the median value for each category.

Because age is one of the strongest risk factors for erectile dysfunction^15,26,27^ and modifies associations of diet^28^ and lifestyle factors^5,29,30^ with erectile dysfunction, we conducted a priori stratified analyses by age and reported primary results within age strata. We conducted a sensitivity analysis without adjustment for incident CVD during follow-up. We also examined whether presence of CVD risk factors modified associations within strata of age by including a cross-product term between the continuous dietary index score variable and the potential effect modifier in our multivariable model. We tested for modification by smoking status (never vs current or past), BMI (<25, 25-29.9, or ≥30), history of hypertension (yes or no) or hyperlipidemia (yes or no), and physical activity (<8.3 vs ≥8.3 total MET-hours/week, corresponding to Department of Health and Human Services guideline recommendations for ≥150 minutes of moderate or ≥75 minutes of vigorous physical activity per week^31^). *P* values were 2-sided, and *P* < .05 was considered statistically significant. For interaction tests, we used a threshold of *P* < .0017 (.05 / 30) based on Bonferroni correction. All analyses were completed in February 2020 using SAS statistical software version 9.4 (SAS Institute).

## Results

Among 21 469 men (mean [SD] age, 62 [8.4] years) included in the analytic sample, compared with men in the lowest categories of either dietary score index, men in the highest categories had older mean (SD) age (MDS: 61 [8] years vs 63 [8] years; AHEI-2010: 60 [8] years vs 64 [9] years), had lower mean (SD) BMI (MDS: 26.4 [4] vs 25.4 [3]; AHEI-2010: 26.4 [4] vs 25.3 [3]), and were more likely to be physically active (mean [SD]: MDS, 32 [39] MET-hr/wk vs 42 [41] MET-hr/wk; AHEI-2010, 27 [36] MET-hr/wk vs 47 [43] MET-hr/wk), nonsmokers (MDS: 4177 men [53%] vs 3700 men [54%]; AHEI-2010: 2219 men [55%] vs 2280 men [54%]), and use cholesterol-lowering medication (MDS: 728 men [9%] vs 1147 men [17%]; AHEI-2010: 421 men [10%] vs 603 men [14%]). Additional baseline demographic and health-related characteristics are reported for the extreme categories of each dietary index score (Table 1). We observed 968 incident erectile dysfunction cases during 53 245 person-years among men younger than 60 years, 3703 cases during 107 048 person-years among men aged 60 to less than 70 years, and 4793 cases during 72 229 person-years among men aged 70 years or older.

**Table 1.** Baseline Characteristics of 21 469 Men From the Health Professionals Follow-up Study by Extreme Categories of Dietary Index Score

Characteristic	No. (%)			
	Mediterranean diet score^a^		AHEI-2010^b^	
	0-3	6-9	Lowest quintile	Highest quintile
Age, mean (SD), y	61 (8)	63 (8)	60 (8)	64 (9)
BMI, mean (SD)	26.4 (4)	25.4 (3)	26.4 (4)	25.3 (3)
Physical activity, mean (SD), MET-h/wk	32 (39)	42 (41)	27 (36)	47 (43)
Race				
White	7306 (93)	6255 (91)	3704 (92)	3890 (92)
Black	42 (<1)	49 (<1)	30 (<1)	21 (<1)
Asian	77 (1)	134 (2)	70 (2)	50 (1)
Other	7425 (5)	449 (7)	230 (6)	270 (6)
Currently married	6987 (89)	6248 (91)	3560 (88)	3821 (90)
Smoking status				
Never	4177 (53)	3700 (54)	2219 (55)	2280 (54)
Past	3120 (40)	3036 (44)	1469 (36)	1866 (44)
Current	558 (7)	151 (2)	346 (9)	85 (2)
Self-reported disease				
Hypertension	1012 (13)	1087 (16)	539 (13)	629 (15)
Hyperlipidemia	3224 (41)	3395 (49)	1720 (43)	1893 (45)
Depression^c^	323 (6)	229 (5)	153 (6)	135 (5)
Medication use				
Antihypertensive	1640 (21)	1678 (24)	915 (23)	937 (22)
Cholesterol-lowering	728 (9)	1147 (17)	421 (10)	603 (14)
Antidepressant or antipsychotic	479 (6)	306 (4)	258 (6)	196 (5)
Benzodiazepine	189 (2)	168 (2)	111 (3)	97 (2)
α-blocker or 5α-reductase inhibitor	411 (5)	387 (6)	185 (5)	267 (5)
Alcohol, mean (SD), g/d	10 (15)	12 (13)	11 (18)	11 (10)
Calories, mean (SD), kilocalories/d	2084 (627)	1932 (582)	1928 (588)	2081 (612)
AHEI-2010 score, mean (SD)	42 (7)	57 (8)	35 (4)	63 (5)
Mediterranean diet score, mean (SD)	2.1 (1)	6.8 (1)	2.3 (1)	6.5 (1)

Abbreviations: AHEI-2010, Alternative Healthy Eating Index 2010; BMI, body mass index (calculated as weight in kilograms divided by height in meters squared); MET, metabolic equivalent task.

^a^ Higher dietary index score indicates greater adherence to a Mediterranean dietary pattern (total score range, 0-9). Exposures were categorized into previously validated ranges (ie, 0-3, 4-5, and 6-9).

^b^ Higher dietary index score indicates greater adherence to the Healthy Eating Pyramid guidelines from the Department of Nutrition, Harvard T.H. Chan School of Public Health (total score range, 0 to 110). Exposures were defined as quintiles.

^c^ Based on self-reported depression on the 2004 questionnaire, the first year this question was asked.

Table 2 displays the associations between MDS and incident erectile dysfunction, stratified by age. Compared with men in the lowest category of MDS, men in the highest category were less likely to develop erectile dysfunction regardless of age group (age <60 years: HR, 0.78; 95% CI, 0.66-0.92; *P* for trend < .002; age 60 to <70 years: HR, 0.82; 95% CI, 0.76-0.89; *P* for trend < .001; age ≥70 years: HR, 0.93; 95% CI, 0.86-1.00; *P* for trend = .04) (Figure, A). This inverse association was greatest among men younger than 60 years (*P* interaction = .003).

**Table 2.** Multivariable-Adjusted Association of Mediterranean Diet Score With Erectile Dysfunction Among Men From the Health Professionals Follow-up Study Stratified by Age

Outcome	Category of Mediterranean diet score^a^			*P* for trend^b^
	0-3 (less healthy)	4-5	6-9 (healthier)	
**Age <60 y**				
Events/person-years, No.	478/22 649	261/16 154	229/14 442	NA
Event rate, per 1000 person-years	21.1	16.2	15.9	NA
Index score, mean (SD)	2.0 (0.9)	4.5 (0.5)	6.8 (0.8)	NA
Age-adjusted model, HR (95% CI)	1 [Reference]	0.76 (0.65-0.88)	0.71 (0.60-0.83)	<.001
Multivariable model, HR (95% CI)^c^	1 [Reference]	0.78 (0.67-0.91)	0.78 (0.66-0.92)	.002
**Age 60 to <70 y**				
Events/person-years, No.	1516/40 007	1109/32 350	1078/34 691	NA
Event rate, per 1000 person-years	37.9	34.3	31.1	NA
Index score, mean (SD)	2.1 (0.9)	4.5 (0.5)	6.8 (0.9)	NA
Age-adjusted model, HR (95% CI)	1 [Reference]	0.87 (0.80-0.94)	0.77 (0.71-0.83)	<.001
Multivariable model, HR (95% CI)^c^	1 [Reference]	0.89 (0.82-0.97)	0.82 (0.76-0.89)	<.001
**Age ≥70 y**				
Events/person-years, No.	1517/22 253	1547/23 031	1729/26 945	NA
Event rate, per 1000 person-years	68.2	67.2	64.2	NA
Index score, mean (SD)	2.2 (0.9)	4.5 (0.5)	6.9 (0.9)	NA
Age-adjusted model, HR (95% CI)	1 [Reference]	0.97 (0.90-1.04)	0.92 (0.86-0.98)	.02
Multivariable model, HR (95% CI)^c^	1 [Reference]	0.98 (0.91-1.05)	0.93 (0.86-1.00)	.04

Abbreviations: HR, hazard ratio; NA, not applicable.

^a^ Higher dietary index score indicates greater adherence to a Mediterranean dietary pattern (total score range, 0-9). Exposures were categorized into previously validated ranges (0-3, 4-5, and 6-9).

^b^ *P* value for trend calculated by modeling the median of each quintile.

^c^ Adjusted for age, race, body mass index, smoking, physical activity, hyperlipidemia, hypertension, depression, antidepressant or antipsychotic medication use, benzodiazepine use, α-blocker or 5α-reductase inhibitor use, incident cardiovascular disease or diabetes during follow-up, caloric intake, and marital status.

**Figure.**
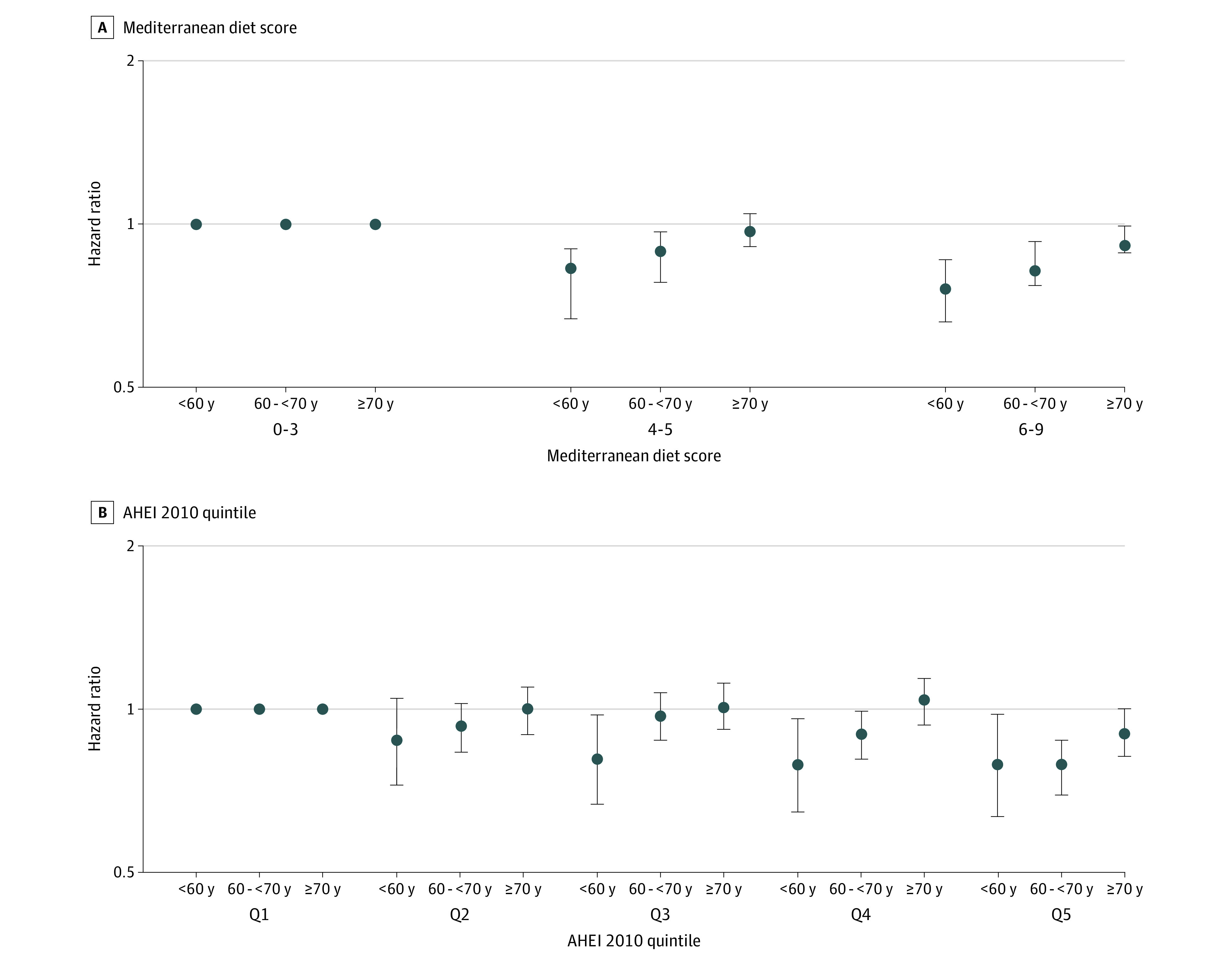
Multivariable-Adjusted Association of Diet Quality Indices With Incident Erectile Dysfunction Stratified by Age AHEI indicates Alternative Healthy Eating Index; Q, quintile.

Table 3 displays the associations between AHEI-2010 score and incident erectile dysfunction, stratified by age. Similar to the MDS, men in the highest quintile of AHEI-2010 score were less likely to develop incident erectile dysfunction compared with men in the lowest quintile regardless of age group (age <60 years: HR, 0.78; 95% CI, 0.63-0.97; *P* for trend = .007; age 60 to <70 years: HR, 0.78; 95% CI, 0.69-0.87; *P* for trend < .001; age ≥70 years: HR, 0.89; 95% CI, 0.81-.99; *P* for trend = .03) (Figure, B). Hazard ratios were also lower among men younger than 60 years compared with older men (*P* for interaction = .0004) and CIs were more likely to exclude 1.0 in lower quintiles of AHEI-2010 index score among younger men.

**Table 3.** Multivariable-Adjusted Association of Alternative Healthy Eating Index 2010 With Erectile Dysfunction Among Men From the Health Professionals Follow-up Study Stratified by Age

Outcome	Quintile of Alternative Healthy Eating Index 2010^a^					*P* for trend^b^
	Q1 (less healthy)	Q2	Q3	Q4	Q5 (healthier)	
**Age <60 y**						
Events/person-years, No.	266/12 299	219/11 754	185/10 959	168/10 281	130/7951	NA
Event rate, per 1000 person-years	21.6	18.4	16.9	16.3	16.4	NA
Index score, mean (SD)	35 (4)	44 (2)	49 (2)	55 (2)	63 (4)	NA
Age-adjusted model, HR (95% CI)	1 [Reference]	0.83 (0.69-0.99)	0.76 (0.63-0.92)	0.71 (0.58-0.86)	0.68 (0.55-0.84)	<.001
Multivariable model, HR (95% CI)^c^	1 [Reference]	0.87 (0.72-1.04)	0.80 (0.66-0.97)	0.78 (0.64-0.95)	0.78 (0.63-0.97)	.007
**Age 60 to <70 y**						
Events/person-years, No.	731/19 212	758/21 748	807/21 925	775/22 552	632/21 612	NA
Event rate, per 1000 person-years	38.0	34.9	36.8	34.4	29.2	NA
Index score, mean (SD)	36 (4)	44 (2)	50 (2)	55 (2)	64 (4)	NA
Age-adjusted model, HR (95% CI)	1 [Reference]	0.91 (0.82-1.00)	0.94 (0.85-1.03)	0.86 (0.77-0.95)	0.71 (0.64-0.79)	<.001
Multivariable model, HR (95% CI)^c^	1 [Reference]	0.92 (0.83-1.01)	0.96 (0.87-1.06)	0.89 (0.80-0.98)	0.78 (0.69-0.87)	<.001
**Age ≥70 y**						
Events/person-years, No.	635/9626	875/13 236	950/13 841	1165/16 500	1168/19 026	NA
Event rate, per 1000 person-years	66.0	66.1	68.6	70.6	61.4	NA
Index score, mean (SD)	36 (4)	44 (2)	50 (2)	55 (2)	64 (5)	NA
Age-adjusted model, HR (95% CI)	1 [Reference]	0.98 (0.89-1.09)	1.02 (0.92-1.12)	1.03 (0.94-1.14)	0.89 (0.81-0.98)	.02
Multivariable model, HR (95% CI)^c^	1 [Reference]	0.99 (0.89-1.09)	1.00 (0.91-1.11)	1.03 (0.93-1.13)	0.89 (0.81-0.99)	.03

Abbreviations: HR, hazard ratio; NA, not applicable; Q, quintile.

^a^ Higher dietary index score indicates greater adherence to the Healthy Eating Pyramid guidelines from the Department of Nutrition, Harvard T.H. Chan School of Public Health (total score range, 0 to 110). Exposures were defined as Q.

^b^ *P* value for trend calculated by modeling the median of each quintile.

^c^ Adjusted for age, race, body mass index, smoking, physical activity, hyperlipidemia, hypertension, depression, antidepressant or antipsychotic medication use, benzodiazepine use, α-blocker or 5α-reductase inhibitor use, incident cardiovascular disease or diabetes during follow-up, caloric intake, and marital status.

When dietary index components were examined separately, higher scores (corresponding to higher intakes) for most healthy components, including vegetables, fruit, legumes, and fish, were associated with lower risk of incident erectile dysfunction. Higher scores (corresponding to lower intakes) for most unhealthy components, including red or processed meat and trans fatty acids, were associated with lower risk of incident erectile dysfunction (Table 4; eTable in the Supplement).

**Table 4.** Multivariable-Adjusted Association of Mediterranean Diet Score Components With Erectile Dysfunction Among Men From the Health Professionals Follow-up Study^a^

Diet	Quintile of Mediterranean Diet Score component intake^b^					*P* for trend^c^
	Q1	Q2	Q3	Q4	Q5	
**Healthy components**^**d**^						
Vegetables, servings/d						
Mean (SD)	1.6 (0.4)	2.4 (0.2)	3.1 (0.2)	3.9 (0.3)	5.8 (1.4)	NA
HR (95% CI)	1 [Reference]	0.95 (0.89-1.01)	0.91 (0.85-0.97)	0.88 (0.83-0.94)	0.85 (0.80-0.91)	<.001
Fruits and nuts, servings/d						
Mean (SD)	1.4 (0.4)	2.2 (0.2)	2.9 (0.2)	3.7 (0.3)	5.1 (1.0)	NA
HR (95% CI)	1 [Reference]	1.00 (0.93-1.06)	0.94 (0.88-1.00)	0.89 (0.83-0.95)	0.84 (0.78-0.90)	<.001
Grains, servings/d						
Mean (SD)	1.4 (0.3)	2.0 (0.1)	2.4 (0.1)	3.0 (0.2)	4.2 (0.8)	NA
HR (95% CI)	1 [Reference]	0.93 (0.87-0.99)	0.98 (0.92-1.05)	0.97 (0.91-1.04)	0.93 (0.87-0.99)	.18
Legumes, servings/d						
Mean (SD)	0.2 (0.1)	0.3 (0.03)	0.4 (0.04)	0.6 (0.1)	0.9 (0.3)	NA
HR (95% CI)	1 [Reference]	0.98 (0.92-1.04)	0.98 (0.92-1.04)	0.92 (0.86-0.98)	0.87 (0.81-0.93)	<.001
Fish, servings/d						
Mean (SD)	0.1 (0.1)	0.2 (0.03)	0.3 (0.03)	0.5 (0.1)	0.8 (0.2)	NA
HR (95% CI)	1 [Reference]	0.97 (0.91-1.03)	0.96 (0.90-1.02)	0.89 (0.84-0.95)	0.86 (0.80-0.92)	<.001
Ratio of polyunsaturated to saturated fat, g/d						
Mean (SD)	0.4 (0.5)	0.5 (0.03)	0.6 (0.03)	0.7 (0.04)	0.9 (0.2)	NA
HR (95% CI)	1 [Reference]	1.00 (0.94-1.08)	1.00 (0.94-1.07)	0.98 (0.92-1.05)	0.91 (0.85-0.97)	.001
**Unhealthy components**^**e**^						
Red or processed meat, servings/d						
Mean (SD)	0.3 (0.1)	0.6 (0.1)	0.8 (0.07)	1.1 (0.1)	1.6 (0.3)	NA
HR (95% CI)	1 [Reference]	1.11 (1.04-1.19)	1.14 (1.06-1.22)	1.21 (1.13-1.30)	1.17 (1.09-1.25)	<.001
Dairy, servings/d						
Mean (SD)	0.8 (0.3)	1.4 (0.2)	1.9 (0.2)	2.5 (0.3)	3.8 (0.9)	NA
HR (95% CI)	1 [Reference]	0.97 (0.91-1.04)	1.02 (0.95-1.09)	1.03 (0.96-1.09)	1.00 (0.94-1.07)	.42
**Moderate components**^**f**^						
Alcohol, g/d						
Mean (SD)	0.1 (0.2)	2.2 (1.2)	6.9 (2.5)	14 (4.3)	31 (12)	NA
HR (95% CI)	1 [Reference]	0.97 (0.90-1.03)	0.96 (0.90-1.03)	0.99 (0.92-1.05)	1.09 (1.01-1.16)	<.001

Abbreviations: HR, hazard ratio; NA, not applicable; Q, quintile.

^a^ Adjusted for age, race, body mass index, smoking, physical activity, hyperlipidemia, hypertension, depression, antidepressant or antipsychotic medication use, benzodiazepine use, α-blocker or 5α-reductase inhibitor use, incident cardiovascular disease or diabetes during follow-up, caloric intake, and marital status.

^b^ Higher dietary index score indicates greater adherence to a Mediterranean dietary pattern (total score range: 0-9). Participants received 1 point for intake of healthy components above the median, 1 point for intake of unhealthy components below the median, and 1 point for alcohol intake between 10 and 50 g/d. Exposures were defined as Q.

^c^ *P* for trend calculated by modeling the median of each Q.

^d^ Lower intake indicates less healthy; higher intake, healthier.

^e^ Lower intake indicates healthier; higher intake, less healthy.

^f^ Moderate intake indicates healthier; higher and lower intake, less healthy.

Smoking status, history of hypertension or hyperlipidemia, BMI, and physical activity did not modify the observed associations. These associations were robust in sensitivity analyses when incident CVD during follow-up was removed as a covariate.

## Discussion

In this cohort study evaluating the association between a healthy dietary pattern measured by 2 dietary index scores and incident erectile dysfunction among men in various age groups, men with the greatest adherence to a Mediterranean or AHEI-2010 dietary pattern were least likely to develop erectile dysfunction. Inverse associations were strongest among men younger than 60 years using AHEI-2010 score; however, men in the highest categories of either dietary index score had the lowest risk of erectile dysfunction in all age groups. These findings suggest that adherence to healthy dietary patterns is associated with lower risk of erectile dysfunction.

To our knowledge, this is the first prospective study that included men without diabetes to evaluate the association between adherence to heathy dietary patterns and incident erectile dysfunction. Our findings are consistent with evidence from prior cross-sectional studies that suggests that men with higher adherence to a Mediterranean dietary pattern are less likely to have prevalent erectile dysfunction^32,33^ and extends prior work by evaluating longitudinal associations between adherence to a Mediterranean or AHEI-2010 dietary pattern and risk of incident erectile dysfunction. Randomized studies have demonstrated that multimodal lifestyle interventions with caloric restriction are effective at improving erectile function among men with erectile dysfunction and significant cardiovascular risk factors.^14,34,35,36,37,38,39^ However, it is difficult to disentangle the independent effect of a healthy dietary pattern from these multimodal intervention studies because obesity and sedentary behavior are associated with increased risk of erectile dysfunction. While we wait for high-quality randomized clinical trials to test whether dietary interventions are effective for preventing or treating erectile dysfunction, our findings support counseling men with low adherence to healthy dietary patterns that they are at higher risk of developing erectile dysfunction compared with men who adhere to healthy dietary patterns.

In a secondary data analysis of men enrolled in randomized clinical trials of dietary interventions, Esposito et al^13^ demonstrated that men with metabolic syndrome and erectile dysfunction randomized to a Mediterranean dietary pattern were more likely to have improvement in erectile function and resolution of erectile dysfunction. The MÈDITA trial^14^ was the first randomized clinical trial to examine the independent effect of a Mediterranean dietary pattern on change in erectile function among men with both normal and abnormal erectile function, to our knowledge, although erectile function was not a primary end point. Among 106 men with newly diagnosed type 2 diabetes in the MÈDITA trial,^14^ those randomized to an energy-restricted Mediterranean diet experienced slower declines in erectile function, measured via the International Index of Erectile Function, compared with men in the energy-restricted low-fat diet group. Our study provides additional evidence that adherence to a healthy dietary pattern may have a protective association against erectile dysfunction beyond the established beneficial effects of weight loss and physical activity on erectile function, particularly among younger men.

A strength of our study is the use of repeated measures of long-term adherence to dietary patterns, which are more aligned with the whole-diet approach to CVD prevention and may be less susceptible to residual confounding due to strongly correlated intake of individual nutrients or food items.^40^ Both MDS and AHEI-2010 consist of components that have been associated with erectile dysfunction or intermediate CVD risk factors in prior studies, which can also provide insight into age-dependent mechanisms of observed associations with erectile dysfunction. Men who report higher fruit and vegetable intake are less likely to have erectile dysfunction in cross-sectional studies^41,42,43^ and short-term interventional studies have demonstrated fruits and vegetables, as well as other antioxidant-rich foods, are associated with beneficial postprandial effects on endothelial function and blood pressure.^28,44,45,46^ Specifically, higher intake of flavonoid-rich foods, a major component of the Mediterranean diet, is associated with lower erectile dysfunction incidence among younger, but not older, men.^28^ Long chain (n-3) fats, most often from fish sources, are associated with lower plasma levels of soluble adhesion molecules and inflammatory markers, which also improve endothelial function.^47^ While our study is the first to our knowledge to evaluate the association between trans fats and erectile dysfunction, trans fats have long been appreciated as a cause of CVD via adverse effects on lipid profiles, proinflammatory changes, and endothelial dysfunction.^48^ This evidence eventually led to the Food and Drug Administration ruling in 2015 to ban trans fats from the US food supply.^49^ Associations between alcohol intake and erectile dysfunction been inconsistently observed in prospective cohort studies^22,41,50,51^; however, interventional studies have demonstrated improvements in inflammatory and lipid biomarkers, as well as cardiometabolic risk factors, with moderate alcohol intake.^52,53^ In addition to the hypothesized associations of individual foods and nutrients, several randomized studies have demonstrated that the Mediterranean dietary pattern as a whole is associated with improved endothelial function, blood pressure, and lipid profiles, as well as decreased inflammatory markers and insulin resistance.^54,55,56^ Furthermore, in the MÈDITA trial,^14^ the beneficial effects of randomization to a Mediterranean diet were also observed among women with diabetes based on improvements in the Female Sexual Function Index; thus, observed associations could be due to differences in sexual function unrelated to erectile function. Although the exact mechanisms have not been elucidated, many components of a Mediterranean or AHEI-2010 diet have been found to be associated with reduced cardiometabolic risk, which is believed to share important biological pathways with erectile dysfunction.

### Limitations

There are several limitations to our observational study. Men were not randomized to their dietary pattern and residual or unmeasured confounding owing to factors that cause unhealthy dietary pattern and erectile dysfunction is possible. However, we considered many widely recognized confounders and took the additional step of stopping dietary exposure updates after a diagnosis of incident CVD during follow-up. Dietary patterns are measured with known error, which can lead to biased results; however, we used well-validated questionnaires to collect long-term dietary intake, and in general, we expect nondifferential measurement error to bias our results toward the null. Additionally, we used a single item question to evaluate erectile dysfunction which could have reduced sensitivity compared with multiple questions; however, a similar single self-report question demonstrated high accuracy (area under the curve, 0.89) compared to urologic examination.^57^

## Conclusions

This cohort study found an inverse association between healthy dietary patterns, such as Mediterranean and AHEI-2010 diets, and risk of developing erectile dysfunction in men. These dietary patterns emphasize the consumption of vegetables, fruits, nuts, legumes, and fish or other sources of long-chain (n-3) fats, as well as avoidance of red and processed meats. These findings suggest that men who are concerned about erectile dysfunction risk should be counseled regarding the potential contribution of their dietary practices. Future randomized clinical trials of healthy dietary patterns in men should include erectile function assessments to determine whether healthy dietary patterns can prevent or reverse erectile dysfunction.

## Notes

Supplement. **eTable.** Multivariable-Adjusted Association of Alternative Healthy Eating Index 2010 Components With Erectile Dysfunction, by Dietary Index Score Component **eFigure.** Study Flowchart
