# Supplementary material for: Association of Diet With Erectile Dysfunction Among Men in the Health Professionals Follow-up Study
Source: JAMA Netw Open. 2020 Nov 13;3(11):e2021701. doi: 10.1001/jamanetworkopen.2020.21701 (PMC7666422; doi:10.1001/jamanetworkopen.2020.21701)
Supplement: Supplement. — eTable. Multivariable-Adjusted Association of Alternative Healthy Eating Index 2010 Components With Erectile Dysfunction, by Dietary Index Score Component eFigure. Study Flowchart [file jamanetwopen-e2021701-s001.pdf]

Supplemental Online Content

Bauer SR, Breyer BN, Stampfer MJ, Rimm EB, Giovannucci EL, Kenfield SA. Association of diet with erectile dysfunction among men in the Health Professionals Follow-up Study. *JAMA Netw Open*. 2020;3(11):e2021701. doi:10.1001/jamanetworkopen.2020.21701

eTable. Multivariable-Adjusted Association of Alternative Healthy Eating Index 2010 Components With Erectile Dysfunction, by Dietary Index Score Component
eFigure. Study Flowchart

This supplemental material has been provided by the authors to give readers additional information about their work.

© 2020 Bauer SR et al. *JAMA Network Open.*

eTable. Multivariable-Adjusted Association of Alternative Healthy Eating Index 2010 Components With Erectile Dysfunction, by Dietary Index Score Component

	Quintile of Alternative Healthy Eating Index 2010 component score[b]					
	**Q1**	**Q2**	**Q3**	**Q4**	**Q5**	***P* for trend**[c]
**Healthy Components**[d]						
Vegetables, score						
Mean (SD)	3.0 (0.7)	4.6 (0.4)	5.9 (0.4)	7.3 (0.5)	9.1 (0.6)	NA
HR (95% CI)	1 [Reference]	0.98 (0.92, 1.05)	0.94 (0.88, 1.00)	0.91 (0.85, 0.97)	0.85 (0.79, 0.91)	<.001
Fruit, score						
Mean (SD)	1.3 (0.5)	2.6 (0.3)	3.8 (0.4)	5.2 (0.5)	7.6 (1.1)	NA
HR (95% CI)	1 [Reference]	0.98 (0.92, 1.05)	0.96 (0.90, 1.03)	0.88 (0.82, 0.94)	0.88 (0.82, 0.94)	<.001
Whole grains, score						
Mean (SD)	1.0 (0.4)	2.0 (0.3)	2.8 (0.3)	3.8 (0.4)	5.7 (1.1)	
HR (95% CI)	1 [Reference]	1.00 (0.93, 1.07)	1.01 (0.94, 1.08)	0.98 (0.91, 1.05)	0.98 (0.91, 1.05)	.40
Legumes and nuts, score						
Mean (SD)	1.2 (0.5)	2.5 (0.4)	3.9 (0.5)	5.5 (0.6)	8.1 (1.1)	NA
HR (95% CI)	1 [Reference]	1.00 (0.93, 1.07)	0.98 (0.91, 1.05)	0.98 (0.91, 1.05)	0.94 (0.87, 1.01)	.06
Polyunsaturated fat, score						
Mean (SD)	2.8 (0.5)	3.8 (0.3)	4.5 (0.2)	5.3 (0.3)	6.6 (0.8)	NA
HR (95% CI)	1 [Reference]	0.97 (0.91, 1.04)	1.01 (0.94, 1.08)	1.03 (0.96, 1.10)	1.04 (0.97, 1.11)	.07
Long-chain (n-3) fats, score						
Mean (SD)	3.4 (1.3)	6.5 (0.7)	8.4 (0.5)	9.6 (0.3)	10 (0)	NA
HR (95% CI)	1 [Reference]	0.99 (0.93, 1.06)	0.93 (0.88, 0.99)	0.95 (0.88, 1.02)	0.88 (0.83, 0.94)	<.001
**Unhealthy Components**[e]						
trans fat, score						
Mean (SD)	6.0 (0.7)	7.2 (0.3)	7.9 (0.2)	8.5 (0.2)	9.3 (0.3)	NA
HR (95% CI)	1 [Reference]	0.96 (0.90, 1.02)	0.95 (0.89, 1.01)	0.95 (0.89, 1.01)	0.87 (0.81, 0.93)	<.001
Sodium, score						
Mean (SD)	1.4 (0.7)	3.4 (0.5)	5.0 (0.5)	6.7 (0.5)	8.6 (0.7)	NA
HR (95% CI)	1 [Reference]	0.99 (0.92, 1.07)	0.97 (0.89, 1.05)	0.96 (0.88, 1.05)	0.95 (0.86, 1.05)	.26
Red or processed meat, score						
Mean (SD)	0.1 (0.1)	1.4 (0.7)	3.7 (0.7)	5.9 (0.6)	8.0 (0.7)	NA
HR (95% CI)	1 [Reference]	1.05 (0.98, 1.12)	0.96 (0.90, 1.03)	0.91 (0.85, 0.98)	0.86 (0.80, 0.93)	<.001
Sugar-sweetened beverages and fruit juice, score						
Mean (SD)	0 (0)	0.4 (0.2)	1.6 (0.6)	4.0 (0.8)	7.3 (1.3)	NA
HR (95% CI)	1 [Reference]	1.02 (0.95, 1.10)	1.05 (0.99, 1.11)	1.06 (1.00, 1.13)	1.03 (0.97, 1.10)	.37

© 2020 Bauer SR et al. *JAMA Network Open.*

eTable Cont'd	***Quintile of Alternative Healthy Eating Index 2010 component score***[b]					
	**Q1**	**Q2**	**Q3**	**Q4**	**Q5**	***P* for trend**[c]
**Moderate Components**[f]						
Alcohol, score						
Mean (SD)	2.3 (0.6)	3.8 (0.5)	5.5 (0.5)	7.7 (0.7)	9.7 (0.4)	NA
HR (95% CI)	1 [Reference]	0.98 (0.91, 1.04)	0.93 (0.87, 0.99)	0.94 (0.88, 1.00)	0.96 (0.90, 1.02)	.20

Abbreviations: HR, hazard ratio; NA not applicable; Q, quintile.

[a] Adjusted for age, race, body mass index, smoking, physical activity, hyperlipidemia, hypertension, depression, antidepressant or antipsychotic medication use, benzodiazepine use, α-blocker or 5α-reductase inhibitor use, incident cardiovascular disease or diabetes during follow-up, caloric intake, and marital status.

[b] Higher dietary index score indicates greater adherence to the Healthy Eating Pyramid guidelines from the Department of Nutrition, Harvard T.H. Chan School of Public Health (component score range: 0 to 10). Exposures were defined as Q.

[c] *P* for trend calculated by modeling the median of each Q.

[d] Lower intake indicates less healthy; higher intake, healthier.

[e] Lower intake indicates healthier; higher intake, less healthy.

[f] Moderate intake indicates healthier; higher and lower intake, less healthy.

© 2020 Bauer SR et al. *JAMA Network Open.*

eFigure. Study flowchart.

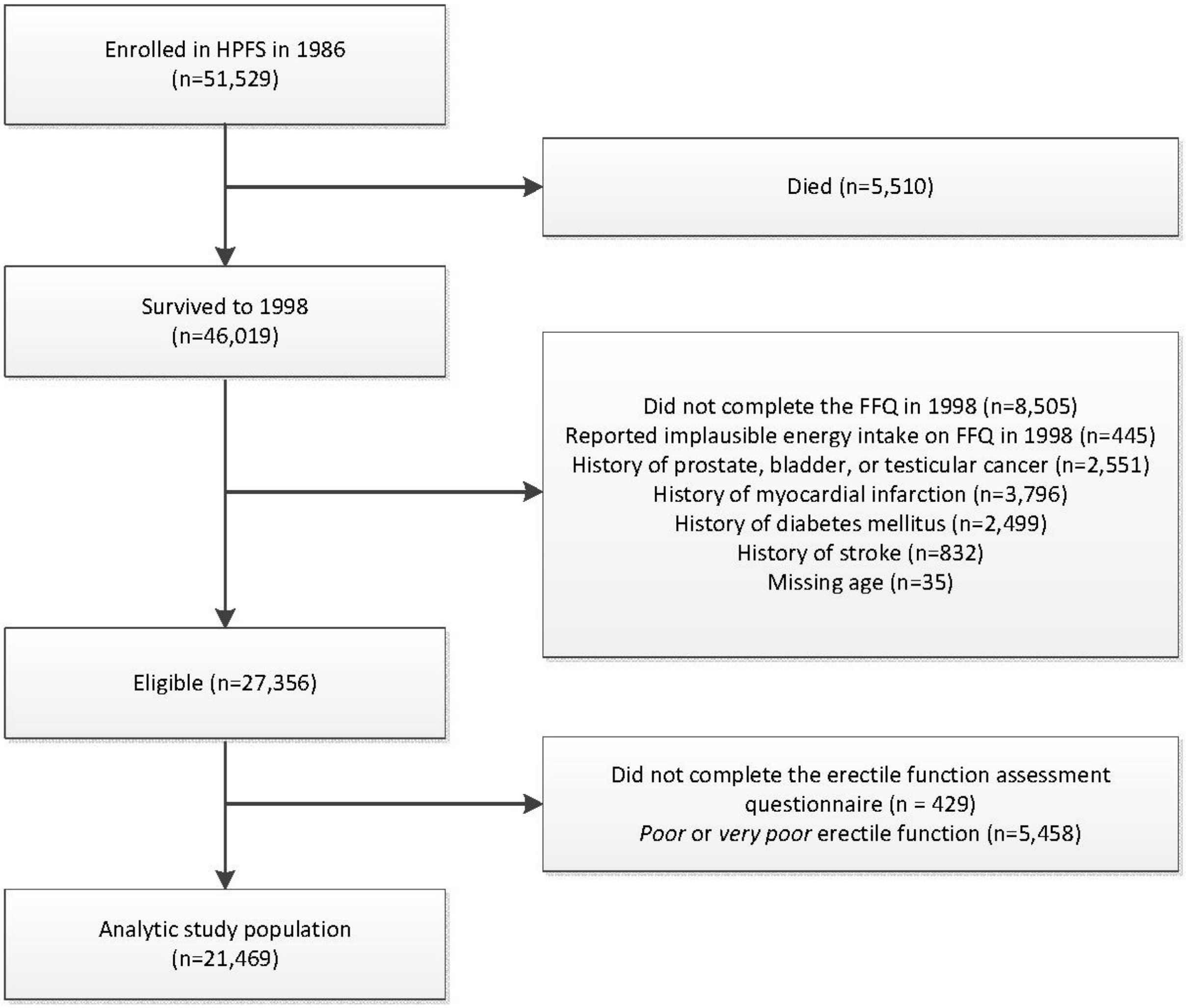

© 2020 Bauer SR et al. *JAMA Network Open.*
